# O-glycan sialylation alters galectin-3 subcellular localization and decreases chemotherapy sensitivity in gastric cancer

**DOI:** 10.18632/oncotarget.13192

**Published:** 2016-11-08

**Authors:** Sofia N. Santos, Mara S. Junqueira, Guilherme Francisco, Manuel Vilanova, Ana Magalhães, Marcelo Dias Baruffi, Roger Chammas, Adrian L. Harris, Celso A. Reis, Emerson S. Bernardes

**Affiliations:** ^1^ Department of Radiopharmacy, Nuclear Energy Research Institute, Radiopharmacy Center, São Paulo, Brazil; ^2^ Department of Center for Translational Oncology Cellular, Biology Group, Center for Translational Oncology, Cancer Institute of the State of Sao Paulo-ICESP, Brazil; ^3^ I3S - Instituto de Investigação e Inovação em Saúde, Universidade do Porto, Portugal; ^4^ IBMC Instituto de Biologia Molecular e Celular, Universidade do Porto, Portugal; ^5^ ICBAS-UP – Instituto de Ciências Biomédicas Abel Salazar, University of Porto, Porto, Portugal; ^6^ Department of Glycobiology in Cancer, IPATIMUP - Institute of Molecular Pathology and Immunology from the University of Porto, Porto, Portugal; ^7^ Department of Clinical, Toxicological and Bromatological Analysis, Faculdade de Ciências Farmaceuticas de Ribeirão Preto, Universidade de São Paulo, Brazil; ^8^ Department of Medical Oncology, Molecular Oncology Laboratories, Weatherall Institute of Molecular Medicine, University of Oxford, Oxford OX3 9DS, UK; ^9^ Department of Pathology and Oncology, Medical Faculty, University of Porto, Portugal

**Keywords:** galectin-3, sialyl-Tn, gastric cancer, glycosylation, chemotherapy resistance

## Abstract

ST6GalNAc-I, the sialyltransferase responsible for sialyl-Tn (sTn) synthesis, has been previously reported to be positively associated with cancer aggressiveness. Here we describe a novel sTn-dependent mechanism for chemotherapeutic resistance. We show that sTn protects cancer cells against chemotherapeutic-induced cell death by decreasing the interaction of cell surface glycan receptors with galectin-3 and increasing its intracellular accumulation. Moreover, exogenously added galectin-3 potentiated the chemotherapeutics-induced cytotoxicity in sTn non-expressing cells, while sTn overexpressing cells were protected. We also found that the expression of sTn was associated with a reduction in galectin-3-binding sites in human gastric samples tumors. ST6GalNAc-I knockdown restored galectin-3-binding sites on the cell surface and chemotherapeutics sensibility. Our results clearly demonstrate that an interruption of *O*-glycans extension caused by ST6GalNAc-I enzymatic activity leads to tumor cells resistance to chemotherapeutic drugs, highlighting the need for the development of novel strategies to target galectin-3 and/or ST6GalNAc-I.

## INTRODUCTION

Modification of cell surface glycosylation is a common feature of cancer cells [1, 2]. Over the last few years, several studies have demonstrated the importance of altered glycosylation in tumor progression and have deepened our understanding in the molecular mechanisms that influence tumor behavior. Because tumor-specific carbohydrate antigens are exclusively expressed by cancer cells and are usually associated with a poor prognosis, they have been commonly used in the clinic as tumor markers [4, 5]. The glycans found in human cells, typically undergo glycosylation in the endoplasmic reticulum-Golgi pathway, and are mainly attached to the protein via an Asn residue (for N-glycans) or, they can be attached by a GalNAc residue in the hydroxyl group of a Ser or Thr residue on the peptide sequence (Tn antigen), for O-glycans [6]. This simplest O-glycan, Tn antigen, can be further converted to a core 1 structure (T antigen) by the addition of a β1,3-galactose extension or to a core 3 structure by the addition of β1,3-GlcNAc. Core 1 structure can be further branched by C2GnT1 to form core 2 that can be further modified to poly-N-acetyllactosamine structures [7].

Shortened or truncated O-glycans seem to be a frequent modification associated with tumor development [8]. These modifications, which include antigen T/Tn, sialyl Thomsen-nouvelle antigen (sialyl-Tn) and sialyl Lewis antigens (sLe) can affect cell surface receptors properties such as, binding, activity and stability; regulate cell-cell and cell-ECM adhesion; or increase cell proliferation and evasion of the immune system [3]. ST6GalNAc-I is the sialyltransferase responsible for the synthesis of sialyl-Tn (sTn) [9], a glycan structure that cannot be further processed, which blocks the posterior elongation of the O-glycan chains [10]. Altered expression of sTn antigen in cancer cells has been shown to be a consequence of multiple mechanisms. Indeed, it was reported that overexpression of ST6GalNAc-I in gastric, breast, prostate and bladder cell lines induced the expression of sialyl-Tn, indicating a fundamental role for this enzyme in sTn biosynthesis [10–13]. Moreover, it has been demonstrated that mutations and loss of heterozygoty of the COSMC gene, which encodes a chaperone protein required for the correct activity of the C1Ga1T1 enzyme (that catalyzes the T antigen), were associated with sialyl-Tn expression in colon and melanoma cell lines and in sTn positive cervical cancer tissue [14–16]. Additionally, hypermethylation of COSMC gene was also found to be associated with increased truncated O-glycans in pancreatic cancer cells [17]. Re-localization of GalNAc-T from the Golgi to the endoplasmic reticulum [18], a reorganization of glycosyltransferase topology [19], and fluctuations in cellular pH [20, 21] were also found to favor shorter glycan chain lengths such as sTn.

Although the association between ST6GalNAc-I and the expression of sTn is still not fully clear, sTn antigen is highly expressed in most gastric [22], colorectal [23], ovarian [24], breast [25] and pancreatic carcinomas [26] whereas no expression is observed in the respective normal tissues. The positive correlation of sTn with carcinoma aggressiveness and poor prognosis has motivated the research on sialyl-Tn role in cancer cell biology. Recently, Radhakrishnan *et al*. showed that truncated O-glycans, such as sTn, could directly induce oncogenic characteristics to tumor cells, including increased proliferation, loss of tissue architecture, disruption of basement membrane adhesion and invasive growth, in a pancreatic model [17]. Likewise, in a gastric cancer model, it was found that sTn antigen was able to induce a more aggressive cell behavior, such as decreased cell–cell aggregation and increased ECM adhesion, migration and invasion [22]. Sialyl-Tn was also found to be responsible for morphological changes, impaired proliferation, and decreased migration on fibronectin and hyaluronic acid strata in a mouse mammary carcinoma cell line [27]. Furthermore, in a murine model, the overexpression of sTn in gastric cancer cells increased their intraperitoneal metastatic ability resulting in shortened survival time of the mice [28]. Recently, ST6GalNAc-I silencing was associated with a reduction in proliferation, migration and invasion of hepatocarcinoma cell through PI3K/AKT/NF-κB pathway [29].

Still, the role of ST6GalNAc-I in chemoresistance remains to be explored. Although the exact mechanism by which sialyl-Tn controls the cancer cell biology is not known, it has been hypothesized that sTn may interfere with the interaction of glycan-binding proteins with glycosylated cell surface proteins, thus promoting tumor progression. So far, this hypothesis has never been studied.

Galectin-3 (gal-3) is a β-galactoside-binding protein that binds a wide array of glycan-containing glycoproteins expressed on the cell surface, thus regulating the activation status of the cell [30]. Similar to ST6GalNAc-I, gal-3 plays an important role in cancer biology. It has been reported that gal-3 expression is increased in tumor cells including breast [31], colon [32], pancreatic [33], thyroid [34] and gastric [35] when compared to normal cells. The alteration of gal-3 expression is correlated with tumor aggressiveness and acquisition of a metastatic phenotype, indicating that gal-3 is able to increase tumor development and influence the progression of the disease.

Gal-3 functions through both intracellular and extracellular mechanisms. Intracellularly, gal-3 was found to have an important role in protecting cells against apoptosis and was reported to decrease BT549 human breast carcinoma cells resistance to cisplatin, anthracycline, adriamycin and 5-FU-induced apoptosis [36]. Upon secretion to the extracellular milieu via a non-classical pathway [37], galectin-3 can bind to cell surface glycans, increasing cell signaling and cell-matrix interactions, for example, through α1β1 integrin [38, 39]. Extracellularly, gal-3 has been shown to preferentially bind to galactose-β1-4-N-acetylglucosamine (LacNAc) units, which can be found in the branches of N- or O-linked glycans [40]. Among the branched N-glycans, the β1,6-GlcNAc-branched product of MGAT5 is the acceptor for additional extension with N-acetyllactosamine units, which is the preferred ligand for galectin-3 [40, 41]. Several groups have reported that N-linked α2-6-sialylation mediated by the ST6Gal-I enzyme, completely blocked recognition by gal-3 [42, 43] whereas α2-3-sialylated glycans were well tolerated [41, 44]. Still, whether the role of other sialylated structures, such as truncated O-glycans like sTn, can contribute to block gal-3 binding to the cell surface has never been studied so far. Moreover, to date, no connection has been made between the intracellular anti-apoptotic function of galectin-3 and the expression of sialylated O-glycans.

In the present study we demonstrated that the overexpression of sTn led to a decrease in gal-3 cell surface binding sites in cancer cells that leads to an accumulation of gal-3 in the intracellular environment, which can account for the chemotherapeutic resistance observed in ST6GalNAc-I-overexpressing tumor cells. Although exogenously added gal-3 did not induce tumor cell death, it showed a potentiating effect on drug-induced cell death. These findings were further validated in human gastric cancer samples, showing that gal-3-binding sites expression correlated negatively with sialyl-Tn levels. Our results suggest that sTn aberrant expression in O-glycans increases gastric carcinoma cells drug resistance by shifting gal-3 subcellular localization.

## RESULTS

### Overexpression of ST6GalNAc-I leads to sialyl-Tn expression and confers resistance to chemotherapeutic drugs in MKN45 gastric cancer cells

**Figure 1:**
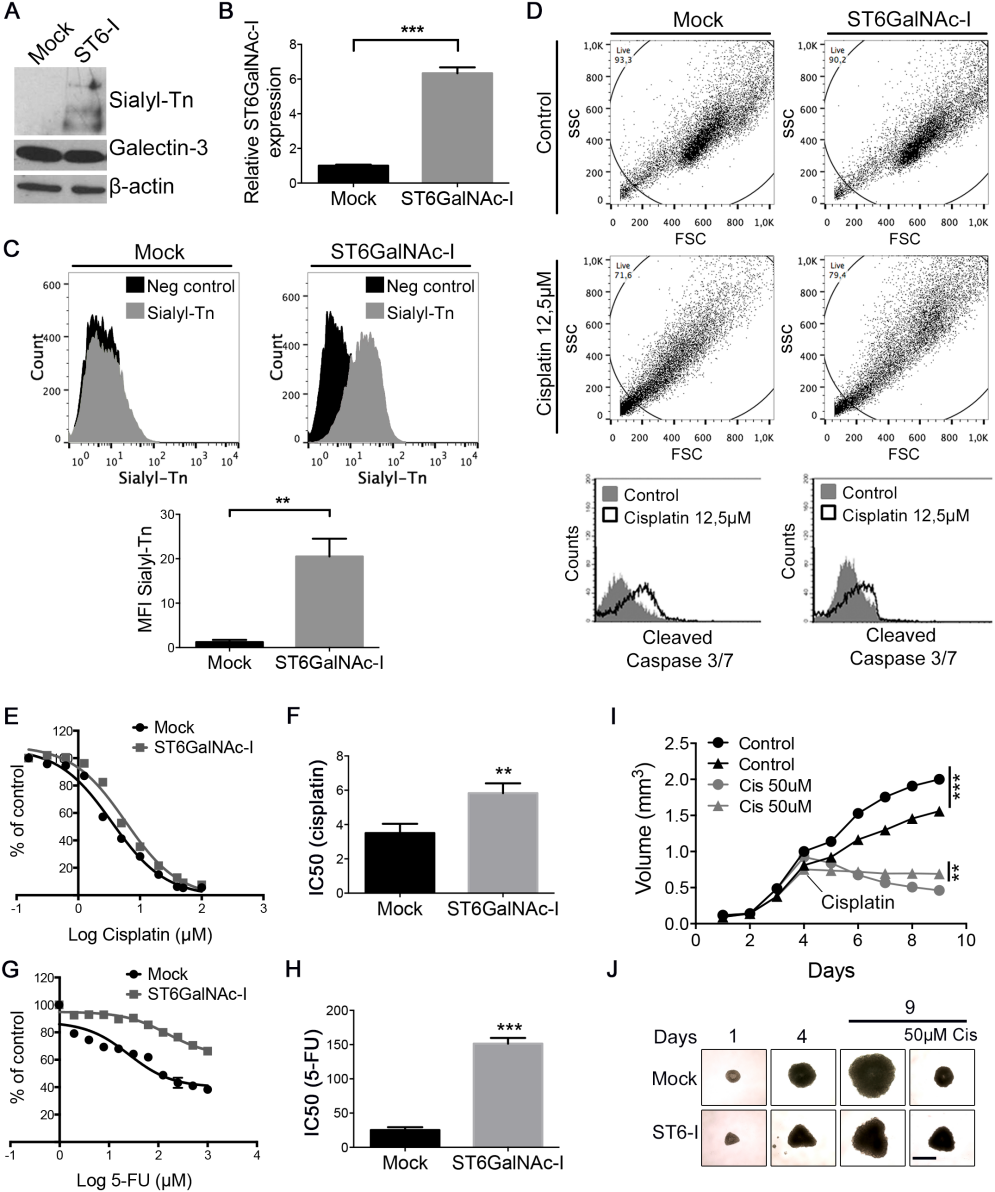
Sialyl-Tn confers chemotherapeutic resistance **A.** Immunoblot of sialyl-Tn and galectin-3 in Mock and ST6GalNAc-I-overexpressing cells. β-actin was used as a loading control. **B.** mRNA levels of ST6GalNAc-I in Mock and ST6GalNAc-I cells. Values were normalized to β-actin. **C.** Flow cytometry histogram and mean fluorescence intensity (MFI) quantification of sialyl-Tn in Mock and ST6GalNAc-I cells. **D.** Flow cytometry dot plot and histogram of caspase 3/7 activation in Mock and ST6GalNAc-I-overexpressing cells undergoing apoptosis by treatment with cisplatin (12.5 μM). **E-H.** SRB assay showing the cells viability under the treatment of (E) cisplatin or (G) 5-FU and determined as a percentage of viable cells relative to control (cells with no drug treatment). **F** and **H.** are the average of IC50 values. **I** and **J.** (I) Growth curve of Mock (circle) or ST6GalNAc-I-overexpressing (triangle) cells spheroids. Cisplatin (50 μM) was added to spheroid culture and day 4 and the spheroid volume was measured until day 9. (J) Representative images of MKN45 spheroids at day 1, 4 and 9, Bar=400 μm). Data are representative or are the mean ± SEM of three independent experiments, **p < 0.01, ***p < 0.001.

The human gastric carcinoma cell line (MKN45), which shows very little expression of sTn [10], was initially transfected with the full length of ST6GalNAc-I or empty vector (Mock). After clonal selection, we found that ST6GalNAc-I-expressing clones #3, #5 and #7 displayed increased ST6GalNAc-I mRNA levels (Supplementary Figure S1A) and sTn antigen levels, as observed by flow cytometry (Supplementary Figure S1B) in comparison with Mock cells. The subsequent experiments were performed with the clone #5 (named ST6GalNAc-I) since it presented the highest increase of sialyl-Tn expression and ST6GalNAc-I mRNA levels (Figures 1A-1C). Mock and ST6GalNAc-I-overexpressing cells were then treated with 12.5 μM of cisplatin for 48h and flow cytometry analysis showed that Mock cells exhibited increased activation of caspase-3/7 in response to cisplatin in comparison with ST6GalNAc-I-overexpressing cells (Figure 1D). Treatment of cells with increasing doses of cisplatin or 5-fluorouracil (5-FU) revealed that ST6GalNAc-I-overexpressing cells were more resistant to the cytotoxic effect of cisplatin and 5-FU than Mock cells, with IC50 values of 5.8 μM (cisplatin) and 152 μM (5-FU) for ST6GalNAc-I-overexpressing and 3.5 μM (cisplatin) and 25 μM (5-FU) for Mock cells (Figures 1E-1H). Additionally, Mock or ST6GalNAc-I-overexpressing cells were grown into spheroids, which replicate important features of tumors *in vivo*, and treated with cisplatin once spheroids reached approximately a volume of 0.5 mm^3^. Although Mock cells formed significantly bigger spheroids than ST6GalNAc-I-overexpressing cells, Mock spheroids presented a significant volume reduction in the presence of cisplatin compared to ST6GalNAc-I-overexpressing spheroids (Figure 1I and 1J). These results indicate that ST6GalNAc-I-overexpressing cells are more resistant to chemotherapeutic drugs than Mock cells.

### Sialyl-Tn expression induces galectin-3 shift into the intracellular compartment

**Figure 2:**
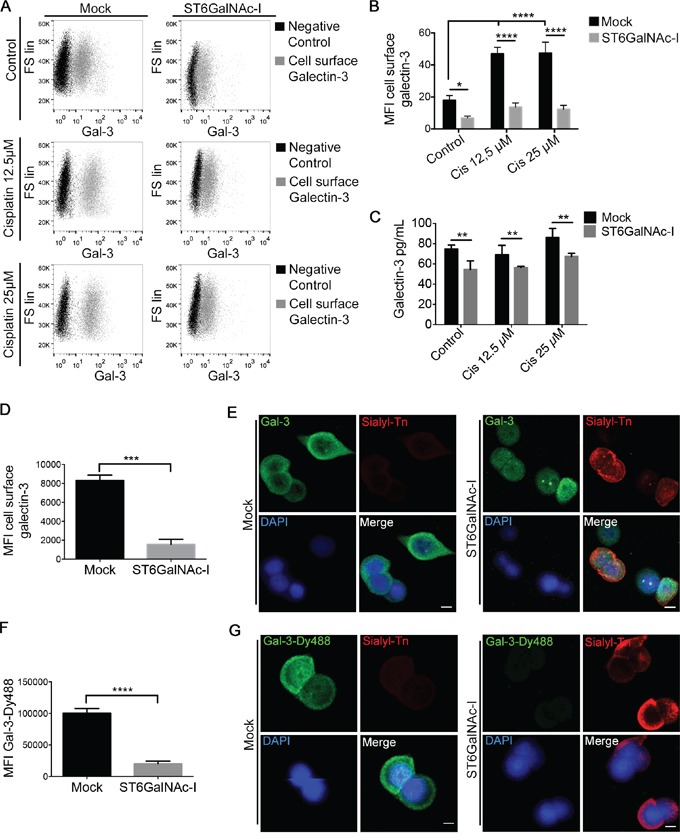
Sialyl-Tn inhibits galectin-3 binding to cellular surface **A** and **B.** (A) Flow cytometry analysis of Mock and ST6GalNAc-I-overexpressing cells stained with anti-galectin-3 antibody after cultured in the presence of cisplatin (12.5 μM and 25μM) and (B) mean fluorescence intensity (MFI) due to galectin-3 staining. **C.** Quantification by ELISA of galectin-3 in culture supernatants of Mock and ST6GalNAc-I-overexpressing cells treated with cisplatin (12.5 μM and 25 μM, as indicated) for 48h. **D.** Mean fluorescence intensity (MFI) of cell surface galectin-3 in Mock and ST6GalNAc-I-overexpressing cells. **E.** Representative immunofluorescence images of Mock and ST6GalNAc-I-overexpressing cells stained with anti-galectin-3 and anti-sialyl-Tn antibodies (Bar=5 μm). **F.** Mean fluorescence intensity (MFI) of galectin-3-binding sites by using Gal-3Dy488 staining in Mock and ST6GalNAc-I-overexpressing cells. **G.** Representative immunofluorescence images of Mock and ST6GalNAc-I-overexpressing cells stained with Gal-3-Dy488 and anti-sialyl-Tn antibody (Bar=5 μm). Data are representative of three independent experiments (A, D-G), or are the mean ± SEM (B and C), *p<0.05; **p<0.01; ****p<0.0001. See also Figure S1.

We next observed that Mock cells treated with cisplatin displayed increased cell surface and supernatants levels of gal-3 (Figures 2A-2C) in comparison with ST6GalNAc-I-overexpressing cells. By flow cytometry, ST6GalNAc-I-overexpressing cells presented reduced levels of cell surface gal-3 in comparison to Mock cells (Figure 2D). These results were confirmed by immunofluorescence microscopy (Figure 2E) and the same findings were observed for ST6GalNAc-I low-expressing clones (Supplementary Figure S1C). We next incubated cells with Gal-3-Dy488 in the presence or absence of lactose (a competitive gal-3 CRD inhibitor) and found that ST6GalNAc-I-overexpressing cells displayed a substantial reduction in gal-3-binding sites compared to Mock cells as observed by flow cytometry analysis (Figure 2F and Supplementary Figure S1D) and immunofluorescence microscopy (Figure 2G). Surprisingly, no changes in the total level of endogenous gal-3 were observed between both cells by flow cytometry analysis (Supplementary Figure S1E) or RT-PCR (Supplementary Figure S1F). Taken together, these data indicate that the overexpression of ST6GalNAc-I induces a cell surface to intracellular shift of gal-3 protein.

### Sialyl-Tn expression reduces the availability of galectin-3-binding sites despite the presence of complex-type N-glycans

**Figure 3:**
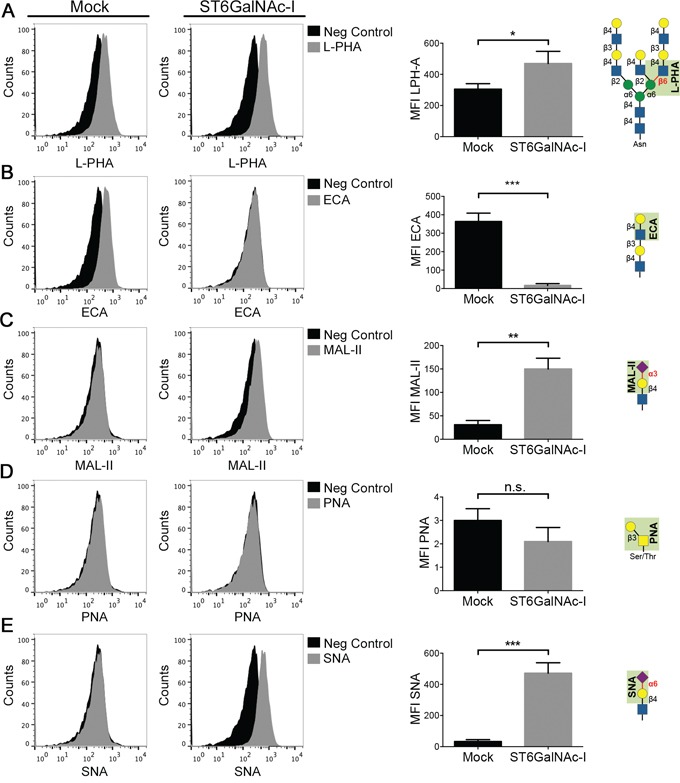
Evaluation of the binding of L-PHA, ECA, MAL-II, PNA and SNA lectins in Mock and ST6GalNAc-I-overexpressing cells Flow cytometry histograms and mean fluorescence intensity (MFI) of Mock and ST6GalNAc-I-overexpressing cells detected with the biotinylated lectins **A.** L-PHA, **B.** ECA, **C.** MAL-II, **D.** PNA and **E.** SNA (grey solid) or with Cy5-conjugated streptavidin alone (filled black). Data are representative images of three independent experiments or are the mean ± SEM, n=3. *p < 0.05, *p < 0.01, ***p < 0.001.

We next evaluated the glycosylation signature of Mock and ST6GalNAc-I-overexpressing cells using a panel of plant lectins that recognize specific glycan structures, including those that are relevant for gal-3 binding. We found that ST6GalNAc-I-overexpressing cells presented increased binding to L-phythemagglutinin (L-PHA), which recognizes tri- and tetraantennary complex-type *N*-glycans, in comparison to Mock cells (Figure 3A). The binding of ECA lectin (*Erythrina Cristagalli*) to unsialylated terminal galactosyl (β-1,4) N-acetylglucosamine was decreased in ST6GalNAc-I-overexpressing cells (Figure 3B), while the binding of MAL-II (*Maackia amurensis* agglutinin), which recognizes α2-3-sialic acid linkages, was increased in ST6GalNAc-I-overexpressing cells (Figure 3C). No difference in the binding of PNA lectin (*Arachis hypogaea*, that recognizes galactosyl (β-1,3) N-acetylgalactosamine present in O-glycans), was found between Mock and ST6GalNAc-I-overexpressing cells (Figure 3D). We also observed that *Sambucus nigra* agglutinin (SNA), a lectin that recognizes α2-6-linked sialic acid present in N-glycans, increased its binding to ST6GalNAc-I-overexpressing cells in comparison to Mock cells (Figure 3E). Altogether, our findings indicate that the O-glycan modification provided by ST6GalNAc-I overexpression can decrease gal-3 binding to the cellular surface also by interfering directly or indirectly with other sialyltransferases, which provide additional evidence about the importance of O-glycans sialylation for gal-3 binding.

### Sialyl-Tn expression protects cells from galectin-3-enhancing effect on the anticancer activity of chemotherapeutic drugs

**Figure 4:**
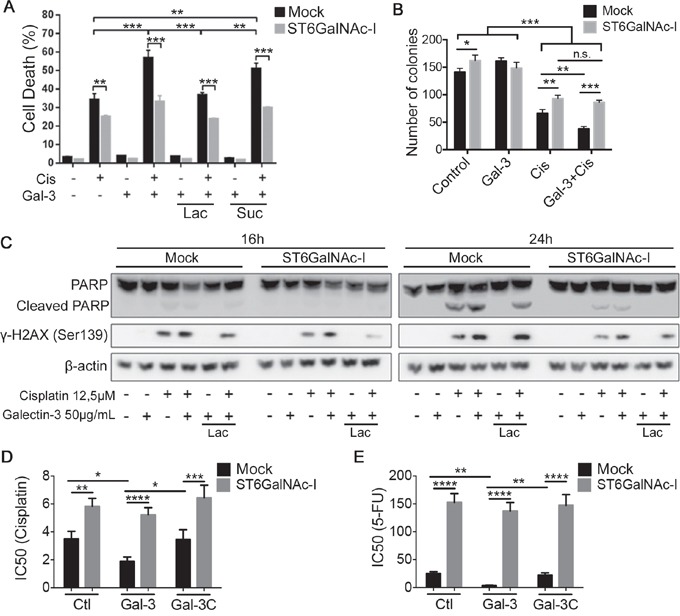
Galectin-3 increases Mock cells susceptibility to cisplatin **A.** Quantification of % of cell death by measuring propidium iodide incorporation, assessed by flow cytomety, in Mock and ST6GalNAc-I-overexpressing cells cultured for 48h with cisplatin and galectin-3 in the presence or absence of lactose. **B.** Clonogenic assay of Mock and ST6GalNAc-I-overexpressing cells after cultured for 48h with cisplatin and galectin-3. **C.** Immunoblot of PARP, cleaved PARP and γ-H2AX, as indicated, in Mock and ST6GalNAc-I-overexpressing cells cultured with cisplatin and galectin-3 in the presence or absence of lactose for 16h and 24h. β-actin was used as a loading control. **D** and **E.** IC50 values for Mock and ST6GalNAc-I-overexpressing cells cultured 48h with (D) cisplatin or (E) 5-FU in the presence or absence of gal-3 or gal-3C. Data are the mean ± SEM, n=3 (A, B, D and E) or are representative of three independent experiments (C). *p < 0.05, **p < 0.01, ***p < 0.001, ****p < 0.0001. See also Figure S2.

Subsequently, we treated Mock and ST6GalNAc-I-overexpressing cells with recombinant human gal-3 (2 μM) and found that gal-3 treatment alone had no effect on cell death (Figure 4A, Supplementary Figure S2A), the ability to form colonies (Figure 4B) or on the cleavage of PARP and phosphorylation of H2AX (γ-H2AX) (Figure 4C) in both cells. However, the combination of both gal-3 and cisplatin led to a significant increase in the percentage of cell death (Figure 4A, Supplementary Figure S2A), reduction in the number colonies (Figure 4B) and increased PARP cleavage and γ-H2AX phosphorylation (Figure 4C) in Mock cells in comparison to cisplatin alone, whereas no changes were observed in ST6GalNAc-I-overexpressing cells. The potentiating effect of gal-3 on cellular death was inhibited by lactose and therefore, dependent on gal-3 carbohydrate binding domain. We next evaluated the survival of Mock or ST6GalNAc-I-overexpressing cells treated with cisplatin or 5-FU in the presence of gal-3 or its N-terminally truncated form (gal-3C). Mock cells incubated with gal-3 displayed a higher susceptibility to the cytotoxic effect of cisplatin (Figure 4D and Supplementary Figures S2B and S2C) or 5-FU (Figure 4E and Supplementary Figures S2D and S2E) as compared to cisplatin treatment alone. Contrastingly, gal-3C did not affect cisplatin or 5-FU cytotoxic effect in Mock cells. Neither gal-3 nor gal-3C had any influence on the cytotoxic effect of cisplatin and 5-FU in ST6GalNAc-I-overexpressing cells (Figure 4D and 4E). Our results demonstrate that although extracellular gal-3 does not directly induce cells death, it potentiates the effect of chemotherapeutic drugs in cells bearing gal-3-binding sites.

### Sialyl-Tn-induced intracellular shift of galectin-3 protects cells from cisplatin induced cell death

**Figure 5:**
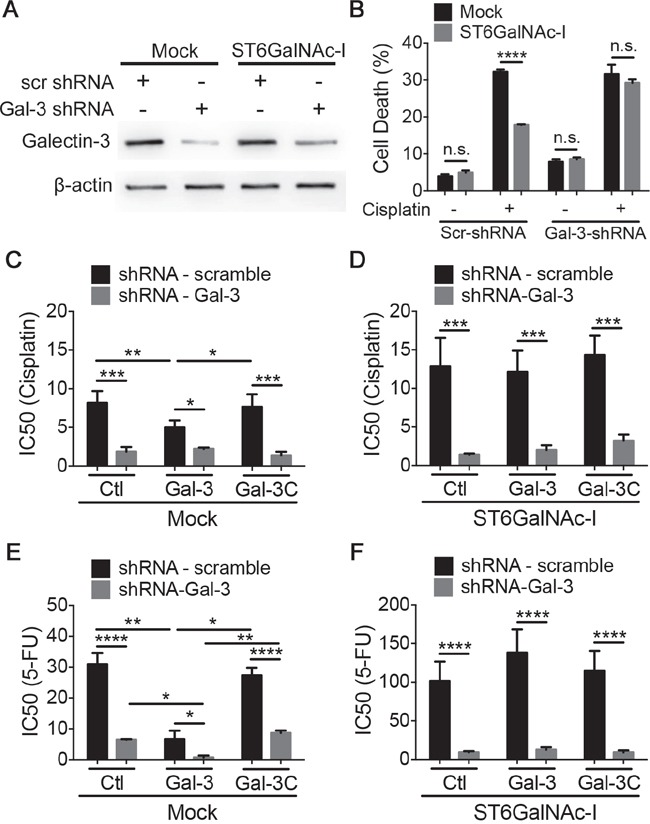
Intracellular galectin-3 protects cells from chemotherapeutic-induced cytotoxicity **A.** Immunoblot of galectin-3 in Mock and ST6GalNAc-I-overexpressing cells transduced with viral particles containing scrambled or shRNA-Gal-3. β-actin was used as a loading control. **B.** Quantification of % of cell death after culturing Mock and ST6GalNAc-I with cisplatin (12.5μM) for 48h. Cells were assayed by propidium iodide and flow cytometry. **C-F.** IC50 values for Mock and ST6GalNAc-I-overexpressing cells transduced with viral particles containing scrambled or shRNA-Gal-3 after culture with cisplatin (C and D) or 5-FU (E and F) and galectin-3 or galectin-3C. Data are representative of three independent experiments (A), or are the mean ± SEM, n=3 (B-F). *p < 0.05, ***p < 0.001, ****p < 0.0001. See also Figure S3.

Since intracellular gal-3 has an important role in protecting cells against apoptosis [45], we subsequently knockdown gal-3 in Mock and ST6GalNAc-I-overexpressing cells using shRNA for gal-3 (Figure 5A). Downregulation of gal-3 had no effect on cisplatin-induced cell death in Mock cells (Figure 5B and Supplementary Figure S3A). On the other hand, ST6GalNAc-I-shRNA-Gal-3 cells presented an increased percentage of cell death, similar to Mock levels, when treated with cisplatin. We further evaluated cisplatin and 5-FU cytotoxicity and showed that gal-3 inhibition significantly increased cisplatin (Figures 5C-5D and Supplementary Figure S3B-S3E) and 5-FU (Figures 5E-5F and Supplementary Figure S3F-S3I) cytotoxicity in both Mock and ST6GalNAc-I-overexpressing cells in comparison to scrambled cells. Incubation with gal-3 increased Mock-scrambled cells susceptibility to cisplatin or 5-FU as compared to cisplatin treatment alone, whereas gal-3C had no effect in cells viability (Figure 5C and 5E). Gal-3 and gal-3C had no effect in the viability of ST6GalNAc-I overexpressing cells (Figure 5D and 5F). Collectively, our results suggest that overexpression of ST6GalNAc-I leads to an increase in cytoplasmic galectin-3 expression, which results in resistance to drug-induced apoptosis.

### ST6GalNAc-I knockdown restores galectin-3-binding sites and sensitizes tumor cell to cisplatin-induced cell death

**Figure 6:**
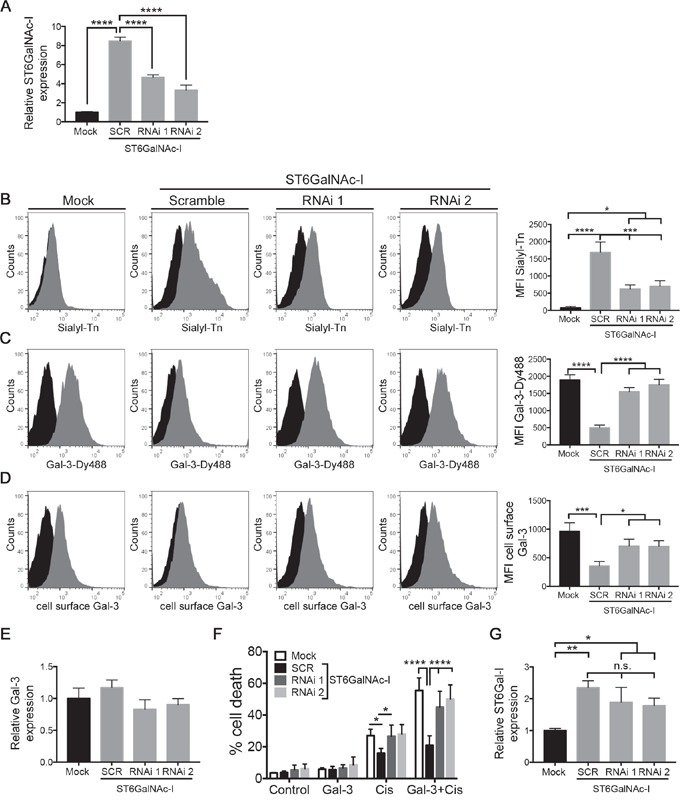
ST6GalNAc-I knockdown increases galectin-3-binding sites and cisplatin-induced cell death **A.** mRNA levels of ST6GalNAc-I in Mock, ST6GalNAc-I and ST6GalNAc-I knockdown cells. Values were normalized to β-actin. **B-D.** Flow cytometry histogram and mean fluorescence intensity (MFI) quantification of (B) sialyl-Tn (grey solid), (C) Gal-3-Dy488 (grey solid) and (D) cell surface gal-3 (grey solid) in Mock, ST6GalNAc-I and ST6GalNAc-I knockdown cells. Negative control: filled black. **E** and **F.** mRNA levels of (E) galectin-3 and (F) ST6Gal-I in Mock, ST6GalNAc-I and ST6GalNAc-I knockdown cells. Values were normalized to β-actin. **G.** Quantification of the % of cell death after culturing Mock, ST6GalNAc-I and ST6GalNAc-I knockdown cells with cisplatin (12.5μM) in the presence or absence of galectin-3 for 48h. Cells were assayed by propidium iodide and flow cytometry. Data are representative of two independent experiments or are the mean ± SEM, n=3. *p < 0.05, *p < 0.01, ***p < 0.001, ****p < 0.0001. See also Figure S4.

To further establish a role for ST6GalNAc-I in gal-3 binding and cisplatin sensitivity, we treated ST6GalNAc-I-overexpressing cells with two different dsRNA (RNAi 1 and RNAi 2). We observed a reduction of 45% (RNAi 1) and 60% (RNAi 2) in ST6GalNAc-I mRNA levels (Figure 6A) and decreased levels of sTn (63% and 58%, respectively) (Figure 6B) in comparison with scramble-treated cells. We next found that ST6GalNAc-I inhibition restored the levels of gal-3-binding sites (Figure 6C) and cell surface gal-3 (Figure 6D) to the levels found in Mock levels. Consistent with our previous results (Supplementary Figure S1E and S1F), no changes in the total mRNA levels of gal-3 were found in Mock, ST6GalNAc-I-overexpressing and ST6GalNAc-I-knockdown cells (Figure 6E). Additionally, we found that cisplatin treatment (alone or in combination with galectin-3) induced higher mortality rate in ST6GalNAc-I knockdown cells in comparison with scramble-treated cells (Figure 6F). The susceptibility to cisplatin-induced cell death in ST6GalNAc-I knockdown cells was comparable to Mock cells. Finally, we evaluated the mRNA levels of ST6Gal-I enzyme, a N-linked α2-6 sialyltransferase, to rule out its interference in blocking gal-3-binding to the cell surface. Even though the levels of ST6Gal-I mRNA were increased in ST6GalNAc-I-overexpressing cells in comparison to Mock cells (Figure 6G), they were not altered between ST6GalNAc-I knockdown and ST6GalNAc-I-overexpressing cells (Figure 6G). These results demonstrate that knocking down ST6GalNAc-I expression is effective in restoring gal-3-binding, gal-3 cell surface expression and in increasing cisplatin sensitivity in tumor cells.

### Sialyl-Tn-expressing tumor xenografts have an increased growth rate and reduced galectin-3-binding sites

**Figure 7:**
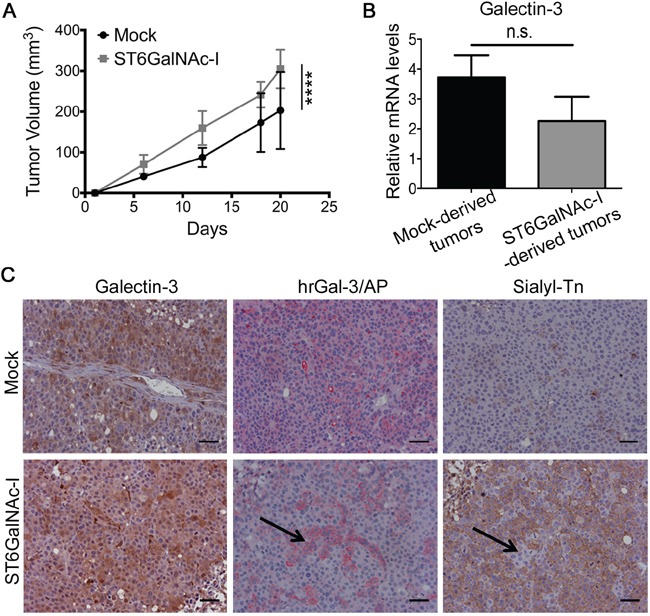
Sialyl-Tn increases tumor growth and decreases galectin-3-binding sites *in vivo* **A.** Tumor growth of Mock and ST6GalNAc-I overexpressing cells in Balb/c nude mice. **B.** mRNA levels of galectin-3 in Mock and ST6GalNAc-I derived tumors. Values are normalized to β-actin mRNA expression. **C.** Immunohistochemical staining of galectin-3, galectin-3-binding sites (hrGal-3/AP) and sialyl-Tn in Mock and ST6GalNAc-I-derived tumors. Black arrows show no co-localization of galectin-3-binding sites with sialyl-Tn. Representative images are shown, bar=20 μm. Data are the mean + SEM (A and B), n=3, or are representative of three independent experiments, five animals per group (C). ****p<0.0001.

We subsequently inoculated Mock or ST6GalNAc-I-overexpressing cells subcutaneously in Balb/c *nude* mice and observed that ST6GalNAc-I-overexpressing tumors had a significantly higher growth rate than Mock-derived tumors (Figure 7A). At the end of the experiment (day 20) tumors were collected and no differences were found between Mock and ST6GalNAc-I-derived tumors with regard to gal-3 mRNA levels (Figure 7B) or protein expression assessed by immunohistochemical staining (Figure 7C). Furthermore, using a human recombinant gal-3 protein fusioned with bacterial alkaline phosphatase (hrGal3/AP) we found that gal-3-binding sites were lower in ST6GalNAc-I-derived tumors in comparison to Mock (Figure 7C). Interestingly, we observed that cells expressing sTn were unable to bind hrGal-3/AP and the opposite was also true (shown by arrows in Figure 7C). Thus, ST6GalNAc-I overexpression confers a selective growth advantage to the tumors, which was associated to a reduced availability of gal-3-binding sites *in vivo*.

### Expression of sialyl-Tn is associated with a reduction in galectin-3-binding sites in human gastric tumors

**Figure 8:**
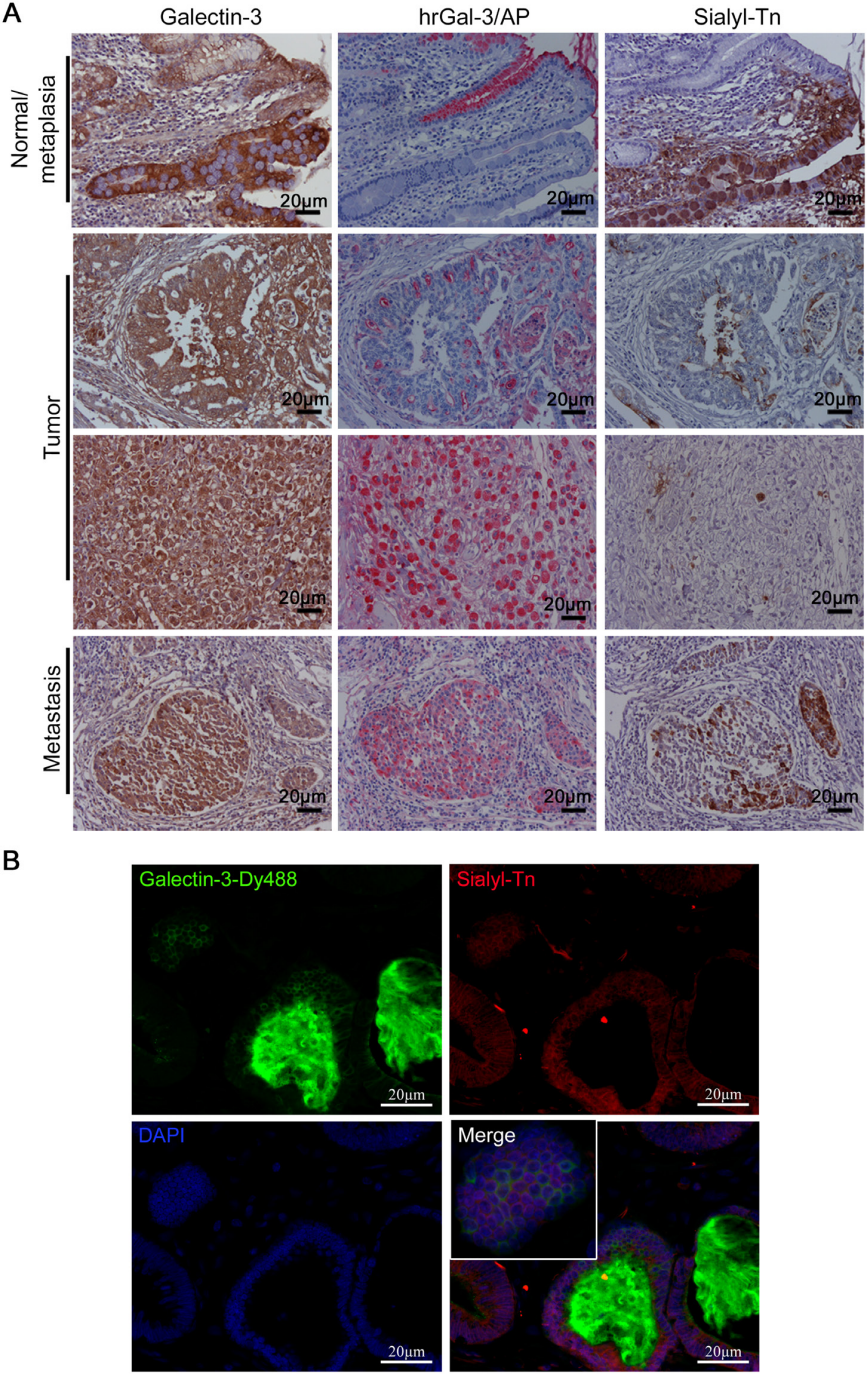
Expression of sialyl-Tn and galectin-3-binding sites in human gastric tumors **A.** Immunohistochemical staining of galectin-3, galectin-3-binding sites (hrGal-3/AP) and sialyl-Tn in human gastric tissue samples. **B.** Immunofluorescence of gastric tumor samples with galectin-3-Dy488, sialyl-Tn and DAPI staining. (A and B) Representative images are shown, bar=20 μm. See also Figure S5.

We further evaluated whether the negative correlation between sTn expression and galectin-3- binding sites could be found in the normal adjacent mucosa, intestinal metaplasia (IM), tumor and associated metastasis in human gastric samples. Gastric samples were classified according to the histological types [24] and the percentage of positive galectin-3, sTn and galectin-3-binding sites stained tumor cells were quantified (Table S1). Galectin-3 expression was found to be strongly expressed in IM and moderately expressed in normal adjacent mucosa (Figure 8A). In the tumor, gal-3 was found to be expressed in 39/40 (97.5%) of the samples and to be present in all 6 gastric cancer-derived metastasis samples. Galectin-3-binding sites were detected in normal adjacent mucosa but not in IM (Figure 8A) and were inhibited by lactose, a gal-3 inhibitor (Supplementary Figure S5). In contrast, sTn was completely absent in normal adjacent mucosa and expressed in IM. In the tumor, gal-3-binding sites were detected in 40/40 (100%) of samples and sTn was expressed in 31/40 (77.5%) of gastric cancer cases, however, gal-3-binding sites and sTn were never co-localized with each other. Also, a negative correlation between gal-3-binding sites and sTn expression was also observed in gastric cancer-derived metastasis, (Figure 8A) that was further confirmed in a double immunofluorescence assay using anti-sTn antibody and human recombinant gal-3 previously labeled with -Dy488 (Gal-3-Dy488) (Figure 8B). These results strongly suggest an important role for sialyl-Tn in reducing galectin-3-binding sites in gastric tumor samples.

## DISCUSSION

The aberrant expression of sialylated proteins is a common feature of cancer cells, which allows cells to escape from the immune surveillance and increases migration and metastasis [8, 22, 25].

Recent studies have provided evidences that α2-6 sialylation of N-linked glycans by the action of ST6Gal-I enzyme can contribute to restrain cell death and to increase therapy resistance in cancer. For example, α2-6 sialylation of Fas and tumor-necrosis factor receptor-1 (TNFR1) by ST6Gal-I inhibited Fas-mediated cell death in colon carcinoma cells [46] and protected macrophages from apoptosis [47]. ST6Gal-I overexpression in ovarian cancer cell was reported to confer resistance to cisplatin treatment, while ST6Gal-I knockdown sensitized cells to cisplatin [48]. Additionally, ST6Gal-I was highly expressed in cisplatin-resistant cells [48] and was associated with increased cancer stem cell resistance to irinotecan and gemcitabine in colon and ovarian cancer cell lines, respectively [49, 50]. Besides chemotherapy, Lee *et al.*, demonstrated that ST6Gal-I expression contributed to colon cancer cells radiation-resistance, still, this effect could be reversed after knockdown of ST6Gal-I or expression of NEU2 sialidase [51, 52]. Expression of other N-linked sialyltransferases, such as ST8Sia I and ST3Gal-I, ST3Gal-II, ST3Gal-III, and ST3Gal-IV were also increased following radiation exposure, however, their role in radiation resistance remains to be investigated [51]. These findings provide several evidences that N-linked sialylation increases resistance to chemo- and radiation therapy, however so far no study has addressed the role O-linked sialylation in chemotherapy resistance. In this study we demonstrate that ST6GalNAc-I overexpression conferred chemoresistance of gastric tumor cells by interfering with gal-3 subcellular localization. To the best of our knowledge, this is the first report to demonstrate the involvement of ST6GalNAc-I-induced sialylation in chemoresistance.

In fact, the specific modification of cell surface proteins with O-glycans has been shown to regulate ligand-receptor binding and cell signaling [53]. For example, MUC1 (mucin 1), CD44 and integrin β1 have been identified as sialyl-Tn carrier proteins, all playing important roles in cell adhesion, migration and chemoresistance [10, 12, 22, 27]. MUC1 has been show to attenuate the apoptotic response to DNA damage conferring resistance to chemotherapeutic drugs such as cisplatin [54]. Similarly, integrin β1 has been shown to block apoptosis induced by cisplatin via ERK and MAP kinase [55, 56] and CD44 molecule has been show to promote PI-3 kinase-mediated oncogenic signaling and cisplatin resistance in cancer cells [57]. Since gal-3 increases the endocytosis of MUC1 [58], integrin β1 [54] and CD44 [59], high levels of sTn on the tumor cell surface might be a mechanism to block gal-3-mediated endocytosis, thus increasing the stability of anti-apoptotic molecules and therefore, chemotherapy resistance.

We also observed that when gal-3 is unable to bind extracellular ligands, because of increased sTn, gal-3 accumulates in the intracellular milieu. This fact might contribute to the chemotherapeutic resistance observed in sTn overexpressing cells. Intracellular galectin-3 has been intensively studied as an anti-apoptotic molecule in response to chemotherapeutic drugs. It was shown that intracellular galectin-3 increased thyroid cancer cells resistance to doxorubicin by activating the PI3K pathway [60]. Recently, Lu *et al*., described that galectin-3 interaction with NF-kB pathway conferred resistance of ovarian cancer cell to carboplatin [61]. Moreover, in lung cancer cells, gal-3 silencing increased drug sensitivity to cisplatin and paclitaxel by regulating the ABCB1 and ABCG2 transporter pumps through β-catenin [62]. In the cytoplasm, galectin-3 heterodiimerzation with Bax was also found to protect cells against doxorubicin-induced apoptosis in thyroid carcinoma cells [63]. Therefore, these and other studies clearly demonstrate that an intracellular accumulation of gal-3 can provide a resistance mechanism to drug-induced cell death.

Here we also observed that tumor cells displaying gal-3-binding sites increased their content of cell surface galectin-3 after cisplatin treatment and presented enhanced susceptibility to chemotherapeutic-induced cell death. One may suggest that increased levels of extracellular galectin-3 can account for the increased sensitivity to chemotherapeutics. Numerous studies have supported the concept that extracellular gal-3 alone is able to promote apoptosis in human leukemia T cell lines [64], human B cell lymphoma [65], neutrophils [66] and in a colon cancer cell model [42]. Though, in this study, exogenously added gal-3 alone did not induce tumor cell death but rather elicited chemotherapeutics-induced cell death in cells displaying gal-3-binding sites.

We believe that when tumor cells present extracellular galectin-3 binding sites, although galectin-3 binding to the cell surface has no effect in the cell viability, its presence in the cell surface may interfere with cellular activation, regulating how cells respond to apoptotic stimulus. Several studies have suggested that galectin-3-glycoprotein lattices are capable of regulating the signaling threshold of a variety of cell surface receptors and to determine receptors residency time at the cell surface [67]. For example, galectin-3 lattice at the cell surface was found to reduce EGF receptor (EGFR) lateral mobility and internalization, and to increase EGFR signaling [68]. On the other hand, some reports have found a direct correlation between EGFR activation and enhanced sensitivity to cisplatin-induced cell death [69–71]. Although extracellular galectin-3 per se has no influence in cellular apoptosis, it's binding to cell surface receptors such as EGFR can sensitize tumor cells to chemotherapeutics. We therefore suggest that the expression of sTn might be a mechanism to protect tumor cells from galectin-3 cell surface binding, thus preventing a cellular response to death-induced stimuli. Nevertheless, the specific regulation of cell surface receptors involved in apoptosis regulation by extracellular galectin-3 remains to be fully investigated and might depend not only on the cell type, but also on which receptors are present and its glycosylation status in a given cell.

Galectin-3 is well known to bind with high affinity to N-acetyllactosamine units, which are commonly found in the N- and O-glycans of glycoproteins. In this study we have shown that despite the fact that ST6GalNAc-I-overexpressing cells presented increased L-PHA-reactive MGAT5-modified N-glycans, preventing the extension of O-linked LacNAc residues with overexpression of ST6GalNAc-I led to reduced gal-3 cell surface binding. Moreover, although ST6GalNAc-I overexpressing cells display increased levels of ST6Gal-I mRNA, we demonstrated that ST6GalNAc-I knockdown had no interference with the expression of ST6Gal-I and was able to restore both gal-3-binding sites and cisplatin resistance. While these data suggest that ST6GalNAc-I overexpression and the consequent increase of sTn expression negatively regulates gal-3 cell surface recognition, we cannot rule out the contribution of others glycosyltransferases in regulating gal-3 cell surface binding. We hope that future studies may help to establish the role and contribution of each individual glyscosyltransferase involved in short O-glycans synthesis and its interference with galectin-3 subcellular localization.

**Figure 9:**
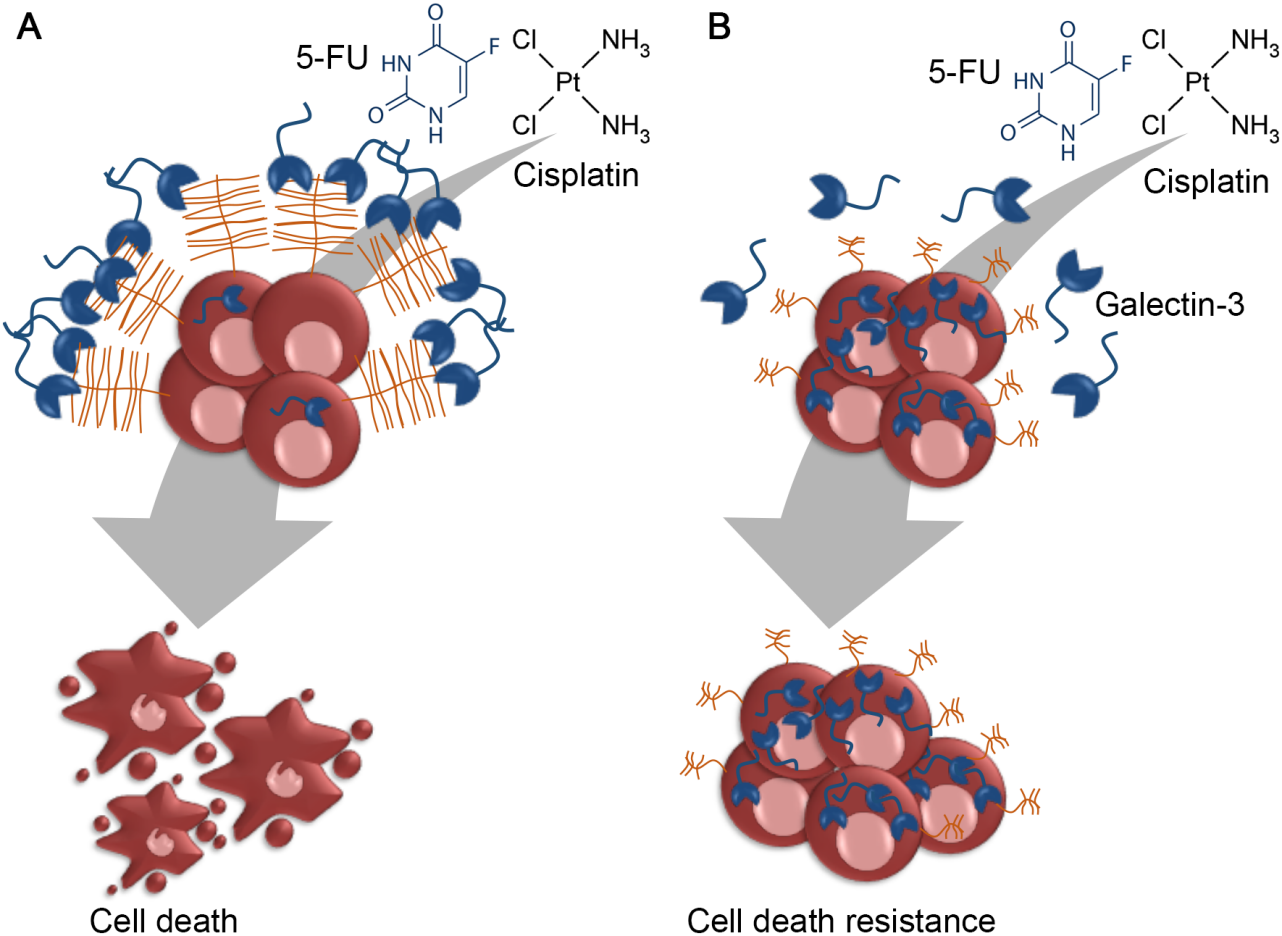
Proposed model for sialyl-Tn mediated resistance to cisplatin **A.** Tumor cells expressing galectin-3-binding sites also express galectin-3 at their cells surface. These cells are more susceptible to extracellular receptors clustering, caused by the action of extracellular galectin-3, thus increasing the sensitivity of tumor cells to cisplatin. **B.** Given that the apoptotic function of gal-3 is related to its subcellular location, tumor cells expressing sTn preferentially accumulate galectin-3 in the cytoplasm. As a consequence, sTn expressing cells are more resistant to chemotherapy and can activate the intracellular anti-apoptotic machinery.

In our study model, we also reported that the gal-3C (which lacks the N-terminal domain responsible for oligomerization), alone or in combination with cisplatin or 5-FU, had no effect on gastric cancer cells viability. Galectin-3C was reported to act as a dominant negative inhibitor of extracellular gal-3 carbohydrate binding and to reduce tumor growth, motility, invasion and angiogenesis through inhibition of galectin-3 [72, 73]. Since the intracellular form of gal-3 accounts for the increased resistance to chemotherapy, it is not surprising that extracellular gal-3 inhibition by gal-3C had no effect on gastric cancer cells viability. Moreover, most of gal-3 extracellular functions are mediated by its ability to oligomerize and crosslink cell surface proteins, therefore, one can assume that the sensitizing effect of gal-3 to chemotherapeutics may be dependent on gal-3-induced crosslink of cell surface receptors. We therefore propose a new mechanism by which sialyl-Tn influences tumor cell resistance to chemotherapeutic drugs (Figure 9). We believe that in a condition where tumor cells display gal-3-binding sites, extracellular gal-3 may form lattices with cell surface anti-apoptotic receptors, increasing their endocytosis and/or restricting their activation by apoptotic stimuli. On the other hand, in the presence of sTn two different mechanism may account for the increased drug resistance: (1) galectin-3 binding is inhibited and cell surface anti-apoptotic receptors can be activated in response to chemotherapeutic treatment; (2) levels of intracellular galectin-3 are increased, which is known to have an anti-apoptotic role.

Thus, the knowledge obtained from this study may open new perspectives in the way we diagnose and treat cancer. Indeed, a careful evaluation of sTn expression and galectin-3-binding sites in tumors may be important to predict patient's response to a specific therapy. In addition, future therapies targeting galectin-3 and/or ST6GalNAc-I enzyme may include the design of RNA interference and/or explore sialyl-Tn expression for a selective drug-delivery approach.

## MATERIALS AND METHODS

### Cell culture

MKN45-Mock and ST6GalNAc-I cells (American Type Culture Collection, Manassas, VALLC [74]) were cultured in RPMI (Gibco, Life technologies, MD, USA) supplemented with 10% of fetal bovine serum (Gibco, Life technologies, MD, USA), 50μg/mL of gentamicin (Gibco, Life technologies, MD, USA) and in the presence of geneticin (300μg/mL) (Sigma). Mycoplasma contamination in cultured cells was excluded by using Lonza Mycoplasma Detection Kit.

### Stable transfections of full-length ST6GalNAc-I

Full length human ST6GalNAc-I vectors were prepared by PCR as previously reported [10]. Gastric carcinoma cell line MKN45 was transfected with plasmid pcDNA3.1/ST6GalNAc-I using the TFx-50 reagent (Promega, Madison, WI). Cells were selected with 0.3 mg/mL of Geneticin for 2 weeks and resistant cells were cloned by the limiting dilution method [10]. ST6GalNAc-I expressing clones were screened by ST6GalNAc-I mRNA expression and by analysis of sialyl-Tn by flow cytometry.

### Flow cytometry

For flow cytometry, cells were harvested and incubated with anti-sialyl-Tn (TKH2 antibody [75]), anti-galectin-3 (M3/38, ATCC TIB166), biotinylated lectins *Erythrina cristagalli* (ECA), *Phaseolus vulgaris* (L-PHA), *Sambucus nigra* (SNA) and *Maackia amurensis* (MAL) (from Vector Laboratories) or biotinylated*-Arachis hypogaea* (PNA) (Sigma-Aldrich). Subsequently cells were washed with PBS, and primary antibodies were detected with anti-mouse-Alexa488 or anti-rat-Alexa488 antibodies, or streptavidin-Alexa488 (all from Invitrogen) for 45 min. Alternatively, cells were incubated with DyLigth488 labelled-hrGal-3. For the cleaved caspase3/7 assay, cells were treated for 48h in the presence or absence of cisplatin (12,5μM). Caspase-3/-7 evaluation was performed accordingly to manufacturer's instructions (Vybrant FAM Caspase-3 and -7 Assay kit, Life Technologies). Analyses were made using the flow cytometer CyAn™ ADP Analyzer from Beckman Coulter. Data were subsequently evaluated with FlowJo vX 0.7 software. For detailed protocol see supplemental experimental procedures.

### Gene expression analysis

Total RNA from cell cultures or tumor tissue was isolated with Tri-Reagent (Sigma) according to the manufacturer's instructions. Complementary DNA (cDNA) was synthesized using the High capacity cDNA RT kit (Applied Biosystems), according to the manufacturer's protocols. Quantitative PCR analysis was performed in triplicate using the SensiMix SYBR No-ROX kit (Bioline). Relative quantification was done using the ΔΔCt method normalizing to GAPDH gene expression (See Table S2 for primer detail).

### ELISA

MKN45-Mock or –ST6GalNAc-I cells were cultured in RPMI supplemented with 10% of fetal bovine serum in the presence of cisplatin (12.5μM or 25μM) for 48h. After this period, the cellular medium was collected and galectin-3 was quantified by ELISA according to the manufacturer's procedure (ELISA galectin-3 Duo Set, R&D Systems).

### Immunofluorescence

Cells or gastric cancer tissues were incubated with anti-sialyl-Tn (TKH2 antibody[75]), anti-galectin-3 (M3/38, ATCC TIB166) or hrGal-3-DyLight488 and detected with anti-mouse-Alexa-546 (Invitrogen) or anti-rat-Alexa488 (Invitrogen), respectively. Nuclei were stained with DAPI (4',6-diamidino-2-phenylindole). Pictures were taken using a fluorescent inverted microscope (Zeiss Axiovert 200M). For detailed protocol see supplemental experimental procedures.

### Immunostaining

Sections from cancer specimens were obtained from patients with mucinous adenocarcinomas undergoing surgery at Hospital S. João, Medical Faculty (Porto, Portugal) between 1991 and 2009. Tissue fragments were immunostained with anti-sialyl-Tn antibody ((TKH2 antibody [75]), anti-galectin-3 (M3/38, ATCC TIB166) or incubated with hrGal-3/AP [76]. The tissue were classified using a 0-to-3 scale: 0 for 0–5% positive tumor cells, 1 for 6–50% positive tumor cells, 2 for >50% positive tumor cells. For detailed protocol see supplemental experimental procedures.

### Statistical analysis

All data are expressed as the mean ± SEM of at least three independent experiments. Statistical analysis including t-test, one-way ANOVA and two-way ANOVA were done using GraphPad Prism 6.0 software. p < 0.05 was considered statistically significant.

## SUPPLEMENTAL DATA


