# Supplementary material for: O-glycan sialylation alters galectin-3 subcellular localization and decreases chemotherapy sensitivity in gastric cancer
Source: Oncotarget. 2016 Nov 8;7(50):83570–87. doi: 10.18632/oncotarget.13192 (PMC5347789; doi:10.18632/oncotarget.13192)
Supplement: Supplementary file 1 [file oncotarget-07-83570-s001.pdf]

O-glycan sialylation alters galectin-3 subcellular localization and decreases chemotherapy sensitivity in gastric cancer

SUPPLEMENTAL DATA

Supplemental experimental procedures

Cell culture

MKN45 cells (American Type Culture Collection, Manassas, VALLC [1]) were cultured in RPMI (Gibco, Life technologies, MD, USA) supplemented with 10% of fetal bovine serum (Gibco, Life technologies, MD, USA) and 50µg/mL of gentamicin (Gibco, Life technologies, MD, USA). Mock and ST6GalNAc-I transfected cells [2] were cultured on the same conditions but in the presence of the selective antibiotic geneticin (300µg/mL) from Sigma. Mycoplasma contamination in cultured cells was excluded by using Lonza Mycoplasma Detection Kit.

Recombinant human galectin-3 and galectin-3C preparation

Galectin-3 and galectin-3C were produced in *Escherichia coli* Bl21/DE3 containing the pET11a plasmid with the human galectin-3 (hrGal-3) coding DNA or the C-terminal domain fragment of galectin-3 (hrGal-3C) (gift from Dr. Nozomu Nishi, Kagawa University, Japan) and purified by affinity chromatography on lactosyl-Sepharose (Sigma) as previously described [3]. Lactose was removed by gel-filtration chromatography on desalting columns and contaminating LPS was eliminated by affinity chromatography on detoxi-gel beads (Pierce). The endotoxin levels were less than 0,1 EU per µg of protein as determined by the LAL method (Lonza). Conjugation of hrGal-3 with DyLight488 was performed accordingly to manufacturer's instructions (Thermo Fischer Scientific). Conjugation of hrGal-3 with VivoTag 680XL was performed according to manufacturer's instructions (Perkin Elmer, Boston, USA).

Flow cytometry

For flow cytometry, MKN45-Mock or MKN45-ST6GalNAc-I cells were harvested and $1x10^6$ cells were incubated with 1% BSA/PBS for 1 hour in at 4ºC. Next, cells were incubated with 1µg of anti-sialyl-Tn (TKH2 antibody [4]), anti-galectin-3 (M3/38, ATCC TIB166), biotinylated lectins *Erythrina cristagalli* (ECA), *Phaseolus vulgaris* (L-PHA), *Sambucus nigra* (SNA) and *Maackia amurensis* (MAL) (from Vector Laboratories) or biotinylated-*Arachis hypogaea* (PNA) (Sigma-Aldrich) for 1 hour. Subsequently cells were washed with PBS, and primary antibodies were detected with anti-mouse-Alexa488 or anti-rat-Alexa488 antibodies, Cy5-conjugated streptavidin for 45 min. After final washing, cells were fixed with 4% paraformaldehyde. For intracellular staining of galectin-3 after the steps described above, cells were permeabilized with saponin 0.5%/PBS and incubated with anti-galectin-3 and, subsequently, with anti-rat-Alexa488 antibodies. Alternatively, cells were incubated with different doses 10µg of DyLigth 488 labelled-hrGal-3 in the presence or absence of lactose (50mM) for 1 hour and fixed in 4% paraformaldeyde.

For the cleaved caspase3/7 assay, cells were treated for 48h in the presence or absence of cisplatin (12,5µM). Caspase-3/-7 evaluation was performed accordingly to manufacturer's instructions (Vybrant FAM Caspase-3 and -7 Assay kit, Life Technologies). For the evaluation of cell death by propidium iodide, the cells previously incubated with cisplatin (12.5µM or 25µM) and hrGal-3 (2µM) in the presence or absence of lactose (50mM) were harvested and stained with propidium iodide 20µg/mL (Invitrogen). Analysis was made using the flow cytometer CyAn™ ADP Analyzer from Beckman Coulter. Data were subsequently evaluated with FlowJo vX 0.7 software.

Evaluation of cell death by propidium iodide

$2x10^5$ MKN45-Mock or MKN45-ST6GalNAc-I cells were seeded overnight in a 6 well plate and then incubated with cisplatin (12.5µM or 25µM) and hrGal-3 (2µM) in the presence or absence of lactose (50mM) for additional 48h. At the end of the experiment, cells were harvested and fixed in ethanol 70%. Cells were then washed 2 times with PBS and incubated with 100µL of PBS containing 0.1% Triton X-100 (Sigma), 200µg/mL RNAse (Sigma), propidium iodide 20µg/mL (Invitrogen) for 30 min in the dark. The propidium iodide fluorescence was analyzed with the flow cytometer CyAn™ ADP Analyzer from Beckman Coulter. Data were subsequently evaluated with FlowJo vX 0.7 software.

Gene expression analysis

Total RNA from cell cultures or tumor tissue was isolated with Tri-Reagent (Sigma) according to the manufacturer's instructions. Complementary DNA (cDNA) was synthesized using the High capacity

cDNA RT kit (Applied Biosystems), according to the manufacturer's protocols. Quantitative PCR analysis was performed in triplicate using the SensiMix SYBR No-ROX kit (Bioline). Relative quantification was done using the ΔΔCt method normalizing to GAPDH gene expression.

Generation of MKN45 galectin-3 knockdown cells

Stable shRNA Mock or ST6GalNAc-I cell line targeting galectin-3 (TRCN0000029305, Sigma) or the negative control (SHC016, Sigma) were generated after co-transfection of 30µg of shRNA-containing plasmids with 15µg pPAX2 and 5µg of pMDG.2 (Addgene) into HEK293t packaging cell line utilizing $CaCl_2$ method. The viral supernatant was recovered and the transduced cells were generated by infection with 2 MOI (multiplicity of infectious units) of shRNA lentiviral particles. On the next day, cells were replaced with fresh medium, and a day later, cells were selected with 1µg/mL of puromycin for 1 week. Galectin-3 knockdown (shRNA-Gal-3) and negative control (scramble) cells were generated and subjected to Western blot to investigate galectin-3 expression.

Western blotting

Cells were lysed in RIPA buffer (Sigma) and 50µg of proteins were separated by Novex NuPAGE SDS-PAGE gel system (Invitrogen) and then transferred overnight to a PVDF membrane (Invitrogen). The membrane was incubated with anti-sialyl-Tn (TKH2 antibody [4]), anti-galectin-3 (M3/38, ATCC TIB166), anti-PARP (cell signaling), anti-p-AKT-ser139 (cell signaling) or anti-γ-H2AX (cell signaling). Anti-β-actin-peroxidase (Sigma) was used as a loading control. Horseradish peroxidase (HRP)-conjugated secondary antibodies (Dako) were detected using the enhanced chemiluminescence (ECL) reagent (GE Healthcare) and images were acquired using ImageQuant (GE HealthCare).

Cell viability assay

2000 cells were seeded in a 96-well plate for 24h. Next, cells were incubated with or without cisplatin (range from 0.09 µM to 50 µM) or 5-FU (range from 0.9 µM to 1 mM) for a period of 72h. Cell mass was determined based on the SRB cell protein stain [5]. After this period, cells were fixed with 10% trichloroacetic acid at 4°C for 1 h. The plates were then washed with distilled water and dried. SRB solution (150µL) at 0.4% (w/v) in 1% acetic acid was added and incubated for 30 min at room temperature. The well plates were then washed with 1% acetic acid and dried. 100µL 10 mM Tris base was added to the wells to solubilize the bound SRB, and absorbance was then read at 515nm on an automated microplate reader (VERSAmax, Molecular Devices, Sunnyvale, CA, USA). Data were analyzed with GraphPad Prism 6.0 software. The experiments were performed at least three times, with each condition plated in triplicate.

Colony formation assay

Cells were plated 500 per well in complete media in six-well plates (Corning, Acton, MA, USA) and allowed to adhere for 24h. The next day cells were treated with cisplatin (12.5µM) in the presence or absence of galectin-3 (2µM). After 24h, cisplatin-containing media was removed, and cells were allowed to form colonies in complete media for 14 days. The colonies were then fixed and stained with methanol containing 1% crystal violet for 1 hour and counted manually.

Sphere formation assay

MKN45-Mock or –ST6GalNAc-I cells were harvested and $5x10^4$ cells/mL were ressuspended in ice-cold complete medium. Matrigel (reconstituted basement membrane, BD Bioscience Bedford, MA) was then added at a final concentration of 2.5% with ice-cold pipette tips to the cell suspension. A volume of 200µL of this suspension was added to each well of a Ultra Low Cluster 96-well plate (Costar 7007) with a round bottom. The spheroid formation was initiated by centrifugation of the plates at 1000g for 10min. The plates were then incubated under standard cell culture conditions and at day 4, 50µM of cisplatin was added to the culture medium and growth was monitored until day 9. Spheroid growth was photographed with an inverted microscope. The spheroid volume was calculated using the ImageJ software and applying the formula (volume (mm^3)=$\pi r^3 4/3$).

Immunofluorescence analysis of cells

MKN45-Mock or MKN45-ST6GalNAc-I cells were plated in a 24 well glass bottom plate at a density of $5x10^4$ overnight. The next day, cells were fixed with methanol. Fixed cells were hydrated with PBS and blocked with 1% of bovine serum albumin (BSA, Sigma) for 1 hour and incubated with anti-sialyl-Tn (TKH2 antibody [4]), anti-galectin-3 (M3/38, ATCC TIB166) or hrGal-3-DyLight488 for 1 hour. After washing, primary antibodies were detected with anti-mouse-Alexa-546 (Invitrogen) or anti-rat-Alexa488 (Invitrogen). Nucleus were stained with DAPI (4',6-diamidino-2-phenylindole) and the coverslips were mounted with Aqua-Poly/Mount medium (Polysciences Inc., Warrington, PA). Pictures were taken using a fluorescent inverted microscope (Zeiss Axiovert 200M).

Immunofluorescence analysis of gastric cancer tissue

Tissue sections were deparaffinized in xylene and rehydrated in serial alcohol dilutions. Tissue sections were then blocked with 1% of bovine serum albumin (BSA,

Sigma) for 1 hour and incubated with anti-sialyl-Tn (TKH2 antibody [4]), or hrGal-3-DyLight488 for 1 hour. After washing, primary antibody were detected with anti-mouse-Alexa-546 (Invitrogen). Nucleus were stained with DAPI (4',6-diamidino-2-phenylindole) and the coverslips were mounted with Aqua-Poly/Mount medium (Polysciences Inc., Warrington, PA). Pictures were taken using a fluorescent inverted microscope (Zeiss Axiovert 200M).

Immunostaining

Tissue sections were deparaffinized in xylene and rehydrated in serial alcohol dilutions. Tissue sections were stained with anti-sialyl-Tn antibody ((TKH2 antibody [4]), anti-galectin-3 (M3/38, ATCC TIB166) followed by a secondary anti-mouse or anti-rat biotinylated antibodies (DAKO). Next, streptavidin-peroxidase (DAKO) was added and color development was done with DAB (DAKO). Nuclei were counterstained with hematoxylin. Tissue samples were washed in PBS and mounted in Vectashield (Vector Laboratories, Inc). Alternatively, tissue samples were incubated with hrGal-3/AP [6] for 2 hours and color development was done with Fast Red TR/ Naphthol AS-MX and TR phosphate (Sigma), according to the manufacturer's instructions. Sections were counterstained with hematoxylin. The coverslips were mounted with Aqua-Poly/Mount medium (Polysciences Inc., Warrington, PA). As a negative control, an assay using lactose to inhibit the carbohydrate recognition domain of galectin-3 was performed. Representative areas of gastric adenocarcinomas were digitalized by digital camera (axioskop Plus, Zeiss, germany). The tissue were classified using a 0-to-3 scale: 0 for 0–5% positive tumor cells, 1 for 6–50% positive tumor cells, 2 for >50% positive tumor cells.

In vivo studies

Balb/c nude mice were bred at the animal facility of FMUSP and all experiments complied with the relevant laws and were approved by local animal ethics committees. Six- to 8-week-old male Balb/c nude mice were implanted subcutaneously with $2x10^6$ MKN45-Mock or MKN45-ST6GalNAc-I cells. Each group consisted of five mice. When tumors reached the size of 1-1.5 cm^3, mice were sacrificed, tumors were excised and fragmented for immunohistochemical staining and mRNA expression analysis. Each animal experiment was repeated at least 3 times.

dsRNA synthesis

The plasmid pcDNA3.1 containing the full length human ST6GalNAc-I was employed as the template for the synthesis of two dsRNA sequences using specific primers containing T7 promoter sequences. For dsRNA 1: Forward primer (5'-3'): TAATACGACTCACTATAGG GAGAAGGCCGCCAACTTCAAATCT, Reverse primer (5'-3'): TAATACGACTCACTATAGGGAGACTGCTGG GGCACTGGAG. For dsRNA 2: Forward primer (5'-3'): TAATACGACTCACTATAGGGAGAGCACTGCTTATG AATCAG

ACGG, Reverse primer (5'-3'): TAATACGACT CACTATAGGGAGAATCCCTTCATCGT GTAGCCG. The dsRNAs were synthetized by *in vitro* transcription using Megascript RNAi kit (Ambion) according to the manufacturer's instructions. Integrity and purity of dsRNA were verified by non-denaturing 1% agarose gel electrophoresis, and the concentration was determined spectrophotometrically. The scramble dsRNA sequence, was generated by the "Random DNA Sequence Generator" (http://www.faculty.ucr.edu/~mmaduro/random.htm). Scramble sequence (5'-3'): GTAAAACGACGGCCAGT GATGAGTCTGGGTGGAGCGCGCCCCATTTATACC GTGAGTAGGGTCGACCAAGAACCGCAAGATGCG TCGGTGTACAAATAATTGTCAACAGACCGTCGTG TTTTGAAAATGGTACCAGCATCTTCGGGCGGTCT CAATCAAGCATGGATTACGGTTGAACTAATACGTA TACTTTGCACGGGTTCACTGCGGTCCGTTCAGAG TCGACCAAGGACACAATCGAGCTCCCATCTGTAT GCTCGACTAACTTGTACCCAACCCCCGGAGCTTG GCAGCTCCTGGGGTATCATGGAGCCTCTGGTTCA TCCCGTGGGATATCAAGCCATGGTCATA GCTGTT.

ST6GalNAc-I dsRNA transfection

ST6GalNAc-I overexpressing cells were transfected at 60% of confluency with two different ST6GalNAc-I specific dsRNAs or negative control (scramble) at a final concentration of 10nM using RNAiMAX reagent (Invitrogen) in Opti-MEM I reduced serum medium (Invitrogen). After 6 hrs, transfection medium was replaced by complete RPMI medium. 24 hrs after transfection, cells were cultured in the presence of cisplatin (12,5µM) in the presence or absence of human recombinant galectin-3 (2µM). Cell death 72h post-transfection was assessed by propidium iodide staining. Alternatively, 72h post-transfection, cells were collected for flow cytometry analysis.

Statistical analysis

All data are expressed as the mean ± SEM of at least three independent experiments. Statistical analysis including t-test, one-way ANOVA and two-way ANOVA were done using GraphPad Prism 6.0 software. $p < 0.05$ was considered statistically significant.

REFERENCES

1. Tamura G, Sakata K, Nishizuka S, Maesawa C, Suzuki Y, Iwaya T, Terashima M, Saito K, Satodate R. Inactivation of the E-cadherin gene in primary gastric carcinomas and gastric carcinoma cell lines. Japanese journal of cancer research : Gann. 1996; 87:1153-1159.
2. Marcos NT, Pinho S, Grandela C, Cruz A, Samyn-Petit B, Harduin-Lepers A, Almeida R, Silva F, Morais V, Costa J, Kihlberg J, Clausen H, Reis CA. Role of the human ST6GalNAc-I and ST6GalNAc-II in the synthesis of the cancer-associated sialyl-Tn antigen. Cancer research. 2004; 64:7050-7057.
3. Hsu DK, Zuberi RI, Liu FT. Biochemical and biophysical characterization of human recombinant IgE-binding protein, an S-type animal lectin. The Journal of biological chemistry. 1992; 267:14167-14174.
4. Jass JR, Allison LM, Edgar S. Monoclonal antibody TKH2 to the cancer-associated epitope sialosyl Tn shows cross-reactivity with variants of normal colorectal goblet cell mucin. Pathology. 1994; 26:418-422.
5. Voigt W. Sulforhodamine B assay and chemosensitivity. Methods in molecular medicine. 2005; 110:39-48.
6. de Melo FH, Butera D, Medeiros RS, Andrade LN, Nonogaki S, Soares FA, Alvarez RA, Moura da Silva AM, Chammas R. Biological applications of a chimeric probe for the assessment of galectin-3 ligands. The journal of histochemistry and cytochemistry : official journal of the Histochemistry Society. 2007; 55:1015-1026.

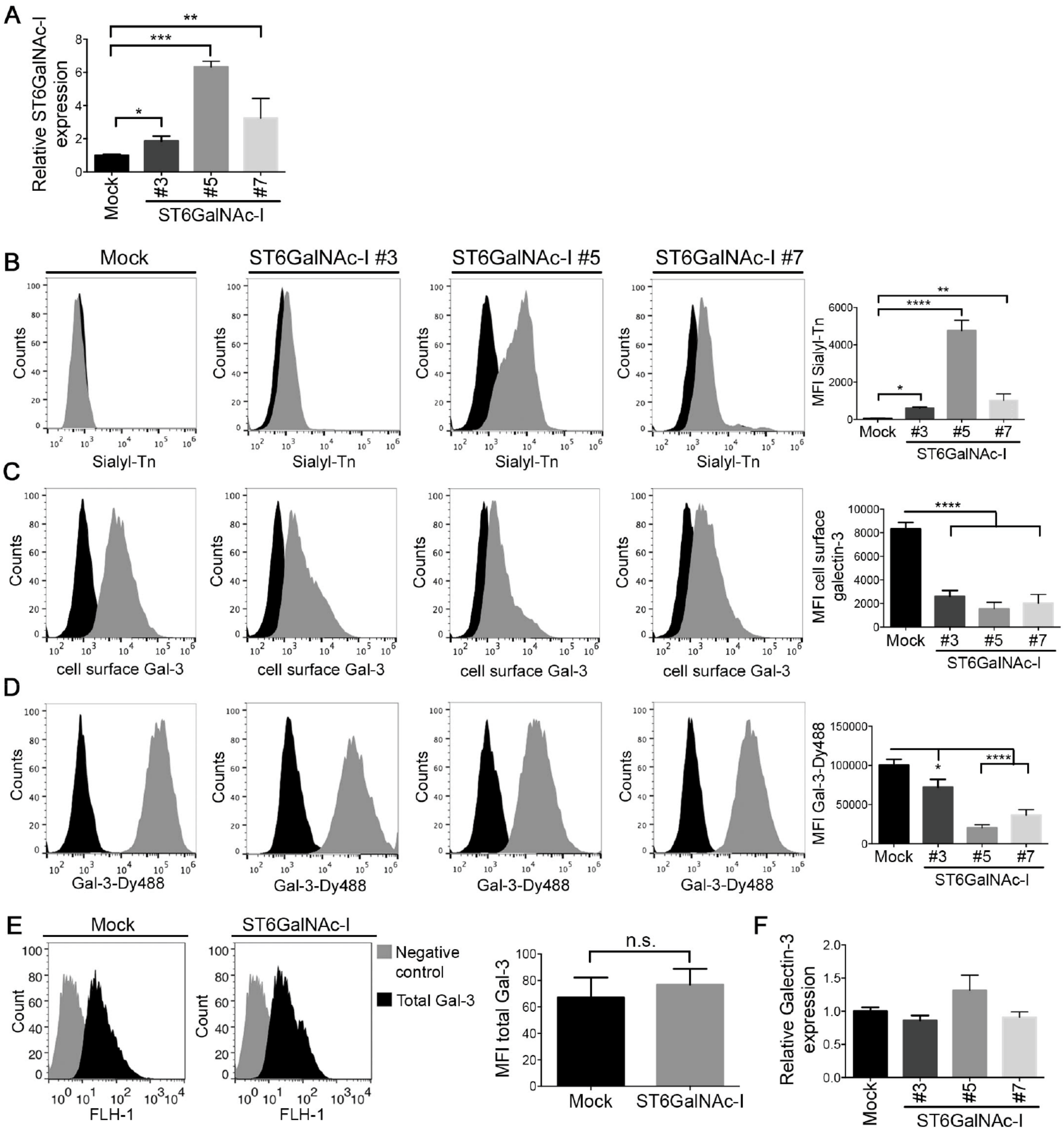

Supplementary Figure S1: ST6GalNAc-I expressing clones present reduced cell surface galectin-3 and galectin-3 binding sites. A. mRNA levels of ST6GalNAc-I in Mock, ST6GalNAc-I expressing clones #3, #5 and #7. Values were normalized to β-actin. **B-D.** Flow cytometry histogram and mean fluorescence intensity (MFI) of (B) Sialyl-Tn (grey solid), (C) cell surface galectin-3 (grey solid) and (C) galectin-3 binding sites (Gal-3-Dy488) (grey solid) in Mock, ST6GalNAc-I clones #3, #5 and #7 cells. Negative control: filled black. **E.** Flow cytometry histogram and mean fluorescence intensity (MFI) of total galectin-3 (grey solid) in Mock and ST6GalNAc-I expressing cells (#5). **F.** mRNA levels of galectin-3 in Mock, ST6GalNAc-I expressing clones #3, #5 and #7. Values were normalized to β-actin. Data are representative images of three independent experiments or are the mean ± SEM, n=3. *p < 0.05, *p < 0.01, ***p < 0.001, ****p < 0.0001.

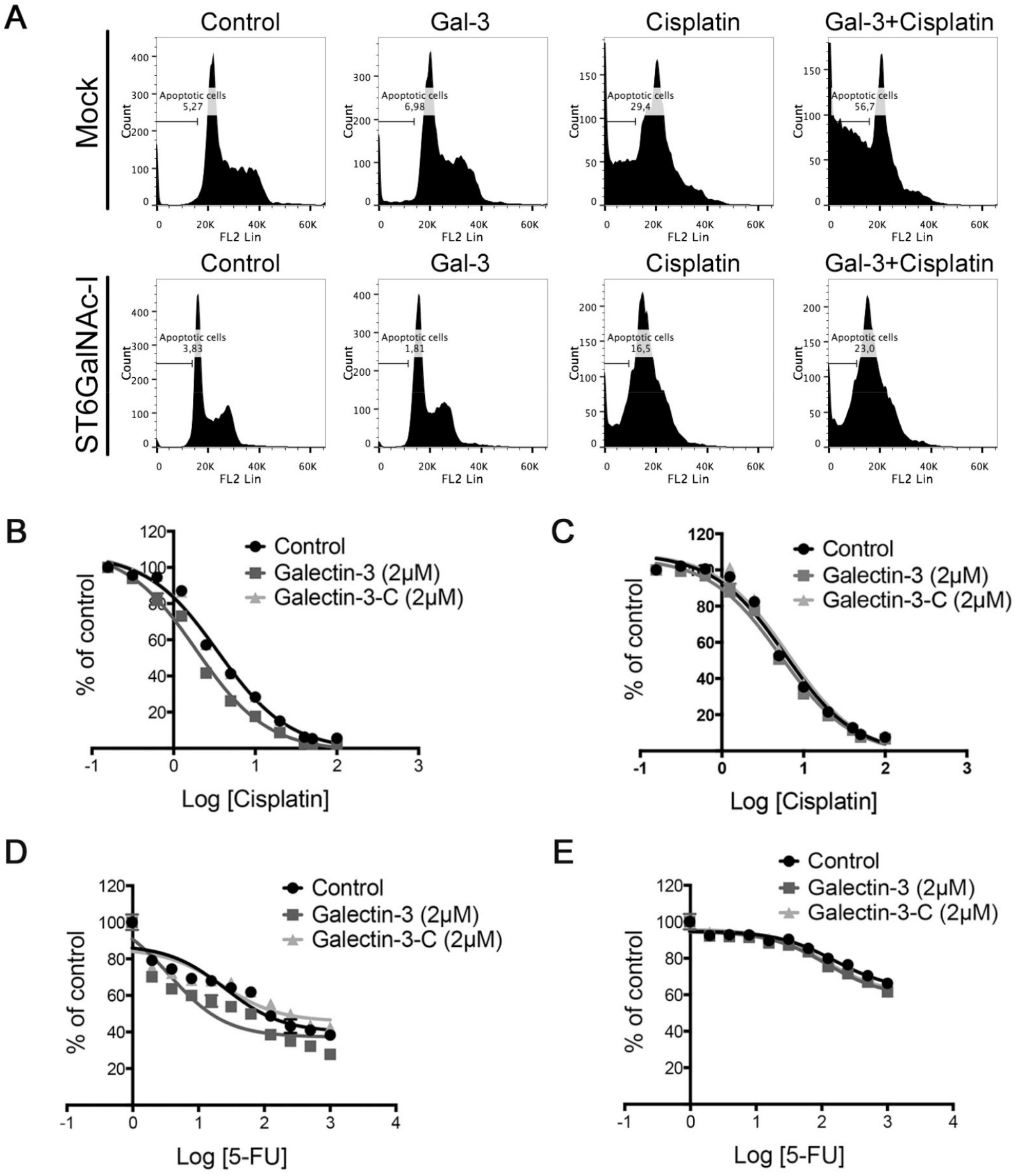

Supplementary Figure S2: Galectin-3 increases cisplatin and 5-FU cytotoxicity in Mock cells. A. Quantification of % of cell death measured by propidium iodide incorporation, assessed by flow cytomety, in Mock and STn-expressing cells cultured for 48h with cisplatin and galectin-3. Representative histograms with the percentage of apoptotic cells are shown. **B-E.** SRB assay showing Mock or STn-expressing cells viability under the treatment of cisplatin (A and B) or 5-FU (C and D) and determined as a percentage of viable cells relative to control (cells with no drug treatment). Data are representative of three independent experiments or are the mean ± SEM, n=3.

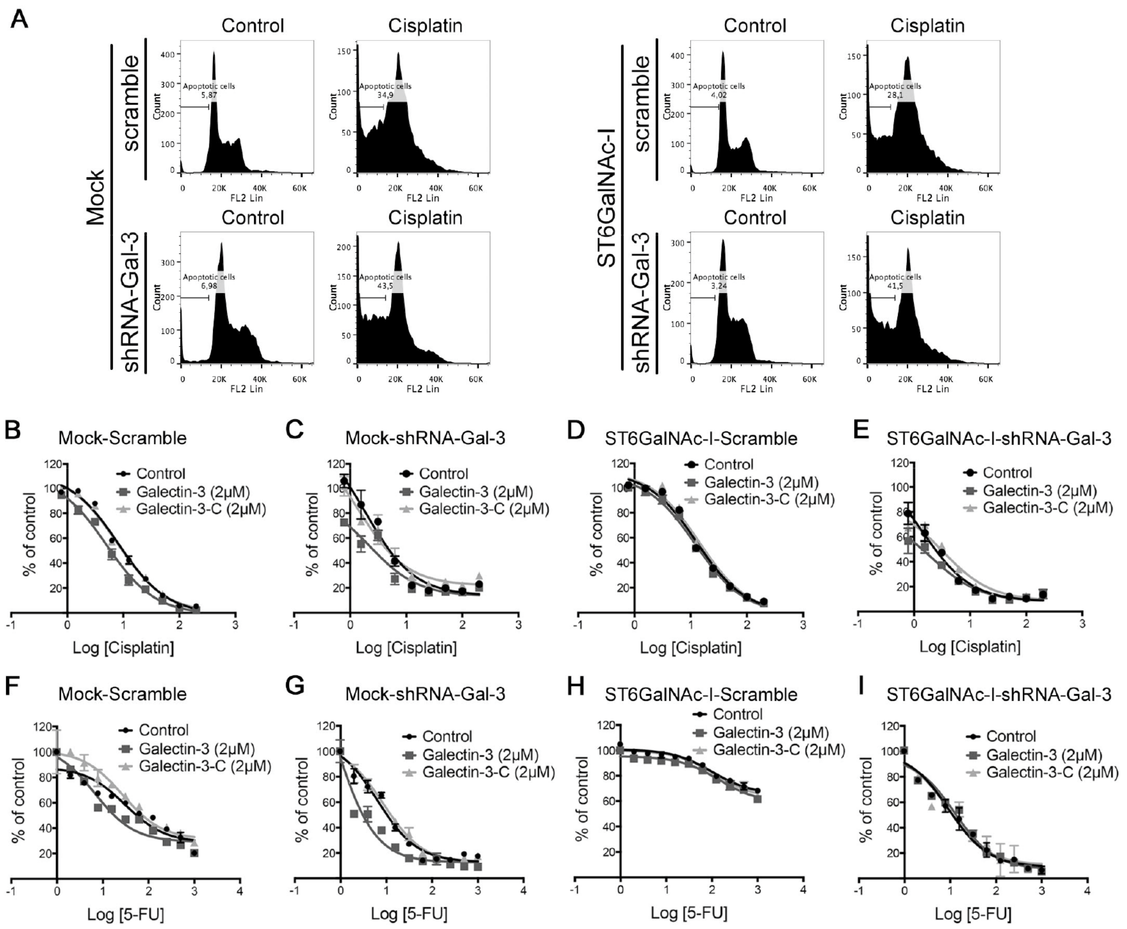

Supplementary Figure S3: Intracellular galectin-3 protects cell from 5-FU induced cytotoxicity. A. Quantification of % of cell death measured by propidium iodide incorporation assessed by flow cytomety in Mock-scramble, Mock-shRNA-Gal-3, ST6GalNAc-I-scramble, ST6GalNAc-I-shRNA-Gal-3 cells after 48h of culture in the presence of 12,5 μM of cisplatin. Representative histograms with the percentage of apoptotic cells are shown. **B-I.** SRB assay showing Mock-scramble, Mock-shRNA-Gal-3, ST6GalNAc-I-scramble or ST6GalNAc-I-shRNA-Gal-3 cells viability under the treatment of cisplatin (B-E) or 5-FU (F-I) and in the presence of galectin-3 or galectin-3C. The indicated concentrations are presented in logarithmic scale and results determined as a percentage of viable cells relative to control (cells with no drug treatment). Data are Data are representative images of three independent experiments or are the mean ± SEM, n=3.

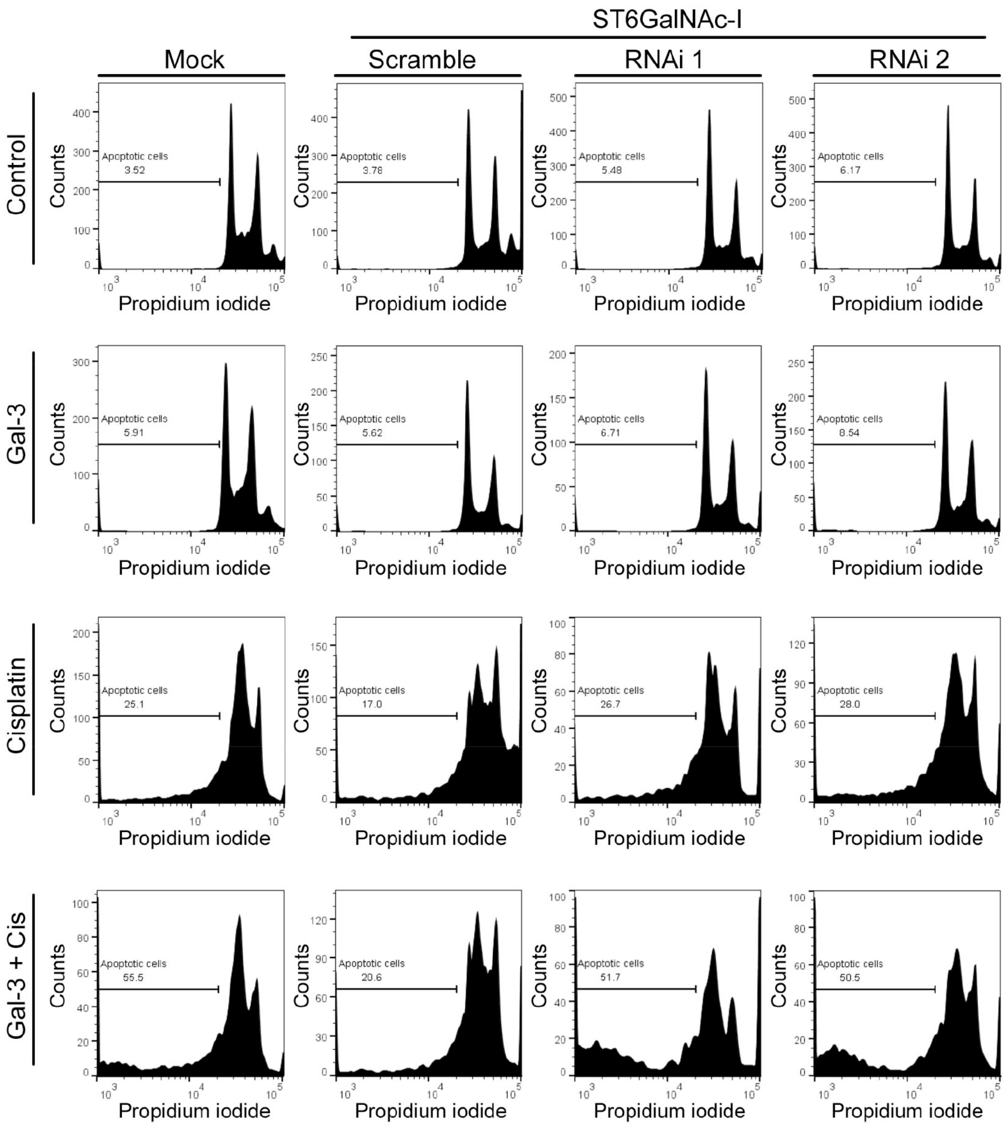

Supplementary Figure S4: ST6GalNAc-I protects cells form cisplatin induced cell death. Quantification of % of cell death measured by propidium iodide incorporation and assessed by flow cytomety in Mock, ST6GalNAc-I-expressing and knockdown cells after 48h of culture in the presence of 12,5 µM of cisplatin +/- galectin-3. Representative histograms with the percentage of apoptotic cells are shown.

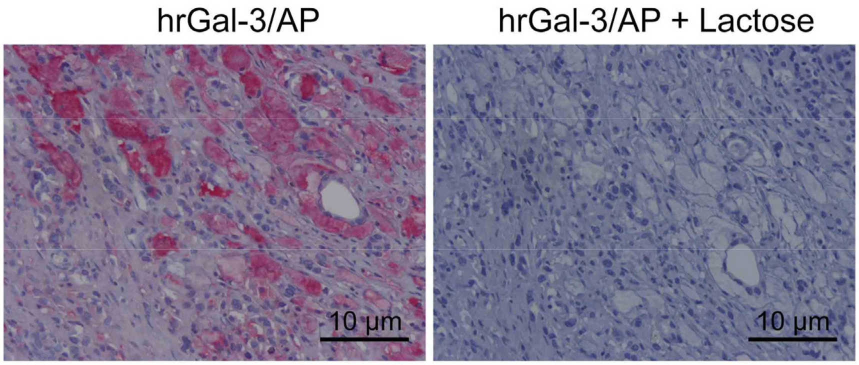

Supplementary Figure S5: Lactose inhibits hrGal-3/AP binding. Immunostaining of human gastric tumor with hrGal-3/AP in the presence or absence of lactose. Bar= 10μm. Representative images are shown.

Supplemental Table S1: Immunohistochemical data

Histological type (n=40)	Number of cases (%)
**Intestinal**	21 (52.5%)
**Diffuse**	11 (27.5)
**Atypical**	8 (20%)
**Galectin-3 expression**	
**Negative**	1 (2.5%)
**<50% of +ve cells**	3 (7.5%)
**>50% of +ve cells**	36 (90%)
**Galectin-3 binding sites (hrGal-3/AP)**	
**Negative**	0 (0%)
**<50% of +ve cells**	3 (7.5%)
**>50% of +ve cells**	37 (92.5%)
**Sialyl-Tn expression**	
**Negative**	9 (22.5%)
**<50% of +ve cells**	20 (50%)
**>50% of +ve cells**	11 (27.5%)

Supplemental Table S2. Primers sequences

Primer	Forward 5' – 3'	Reverse 5' – 3'
ST6GalNAc-I	TCCAAGGGAACACTTGAACCA	GCCTCAGGACCTACAGCAAT
ST6Gal-I	ACGCAGTCCTGAGGTTTAATGG	TCTGTGGTAACCAACTGAGAG
Galectin-3	TGTTTGCAATACAAAGCTGGA	GCAACCTTGAAGTGGTCAGG
α-actin	GCCAGGTCATCACCATTGG	GGTAGTTTCGTGGATGCCACA
